# Supplementary material for: Identification of photoperiod-induced specific miRNAs in the adrenal glands of Sunite sheep (Ovis aries)
Source: Front Vet Sci. 2022 Jul 22;9:888207. doi: 10.3389/fvets.2022.888207 (PMC9354845; doi:10.3389/fvets.2022.888207)
Supplement: Supplementary file 1 [file Data_Sheet_1.ZIP › Supplementary file/miRDeep2 file.zip!/miRDeep2 file/Know_Structure/LP42A1/oar-mir-494.pdf]

miRBase precursor : oar-mir-494
Total read count : 17636
oar-miR-494-5p read count : 31
oar-miR-494-3p read count : 17601
remaining reads : 4

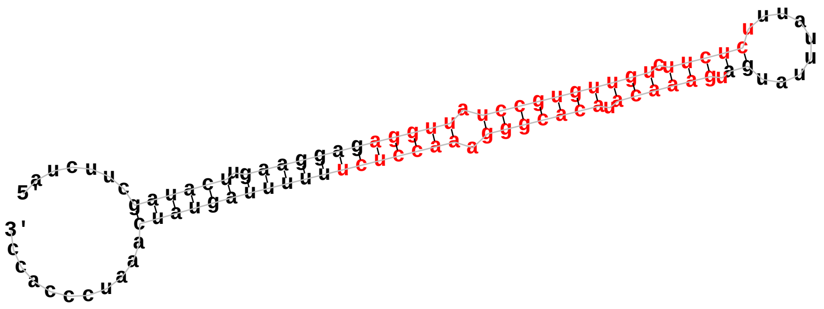

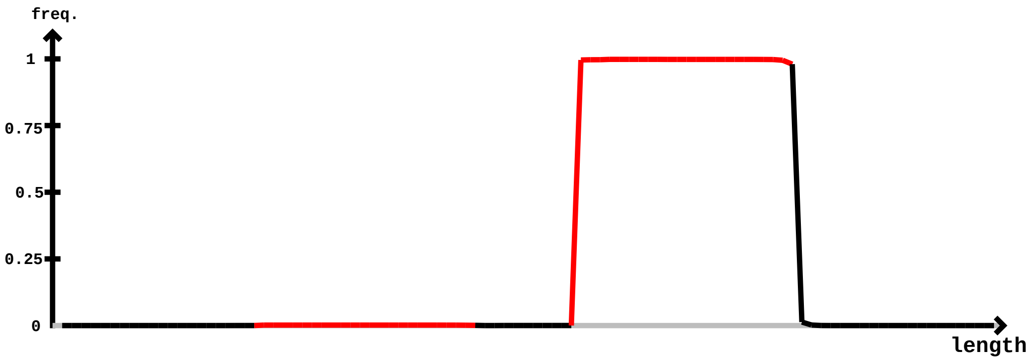

oar-miR-494-3p

oar-miR-494-5p

```
5'- aucuucgauacuugaaggagagguuauccguguugucuucucuuuauuuaugaugaaacauacacgggaaaccucuuuuuuaguaucaaaucccacc   -3'    exp
    ......(((((.(((((((((((.((((((((((.(((((.........)).)))))).))))))).))))))))))))))))..............   reads      mm         sample
    .................gagagguuauccguguugucuu..........................................................   1          0          seq
    ..................agagguuauccguguugucuu..........................................................   1          0          seq
    ....................agguuauccguguugucuucu........................................................   7          0          seq
    ....................agguuauccguguugucuucuc.......................................................   9          0          seq
    ....................agguuauccguguugucuucucu......................................................   14         0          seq
    .............................................auuuaugaugaGacaua...................................   3          1          seq
    .....................................................ugaaacauacacgggaaa..........................   1          0          seq
    .....................................................ugaaacauacacgggaaac.........................   3          0          seq
    .....................................................ugaaacauacacgggaaacc........................   5          0          seq
    .....................................................ugaaacauacacgggaaaccu.......................   48         0          seq
    .....................................................ugaaacauacacgggaaaccuc......................   257        0          seq
    .....................................................ugaaacauacacgggaaaccucu.....................   17035      0          seq
    .....................................................ugaaacauacacgggaaaccucuu....................   195        0          seq
    .....................................................ugaaacauacacgggaaaccucuuu...................   12         0          seq
    .....................................................ugaaacauacacgggaaaccucuuuu..................   3          0          seq
    ......................................................gaaacauacacgggaaaccucu.....................   16         0          seq
    .......................................................aaacauacacgggaaaccucu.....................   3          0          seq
    ........................................................aacauacacgggaaaccucuu....................   1          0          seq
    ........................................................aacauacacgggaaaccucuuu...................   21         0          seq
    ........................................................aacauacacgggaaaccucuuuu..................   1          0          seq
```
